# Remote versus face-to-face delivery of the Group Triple P parenting programme: a feasibility non-randomised trial

**DOI:** 10.1186/s40814-026-01861-3

**Published:** 2026-06-24

**Authors:** Elinor Coulman, Susan Channon, Lauren Copeland, Myrsini Gianatsi, Nina Jacob, Ben Karatas, Stavros Petrou, Rebecca Playle, Michael Robling, Elizabeth-Ann Schroeder, Rory Sheppard, Laura Winney-Jones, Linda Adara, Jeremy Segrott

**Affiliations:** 1 Centre for Trials Research, Cardiff University, Cardiff, UK; 2 DECIPHer, SOCSI, Cardiff University, Cardiff, UK; 3 Nuffield Department of Primary Care Health Sciences, University of Oxford, Oxford, UK; 4 Cardiff Metropolitan University, Cardiff, UK

**Keywords:** Group Triple P, Feasibility, Trial, Parenting, Intervention, Remote delivery

## Abstract

**Background:**

Many well-designed parenting interventions have demonstrated effectiveness in improving outcomes for parents and children. During the COVID pandemic, many group-based parenting interventions were rapidly transferred to remote delivery using online videoconferencing platforms. However, the evidence on the effectiveness of remotely delivered group-based parenting programmes, compared to face-to-face programmes, remains inconclusive. This trial aimed to assess the feasibility of delivering the Group Triple P intervention to parents/caregivers of young children to inform a potential, definitive trial of the effectiveness and cost-effectiveness of Group Triple P delivered remotely compared to face-to-face delivery.

**Methods:**

This study was a feasibility non-randomised trial, with embedded process evaluation. Parents/caregivers of a child (up to 12 years old) were recruited by Local Authority or independent provider research sites and self-selected remote or face-to-face delivery of the Group Triple P intervention. Data were collected at baseline and 16 weeks follow-up. The following feasibility outcomes were assessed: site recruitment, participant recruitment rates and retention at the 16-week follow-up, intervention adherence, fidelity and reach, feasibility of trial processes and outcome measures (including resource use, cost and health-related quality of life) and Local Authority and independent service provider willingness to participate in a definitive trial.

**Results:**

All sites utilised a multi-point participant recruitment strategy, relying on existing pathways to identify families. Only two sites reached participant recruitment targets. Participants’ self-selected remote delivery (23 participants) or face-to-face delivery (19 participants) and baseline demographics, except for education level, were balanced across arms. A 66.7% retention rate was observed at 16 weeks. Most trial processes were acceptable to participants and site staff. However, randomisation was not considered acceptable to site staff or 37.5% of participants, due to the removal of participant choice. The intervention was delivered to high fidelity, except for some intervention adaptations. Participant engagement in the remote arm was hampered by inconsistent use of cameras and little interaction between parents and facilitators.

**Conclusions:**

A number of barriers to using a randomised trial design were identified. Ongoing research should consider a potential non-randomised definitive trial to assess the use of remotely delivered group-based parenting interventions and to overcome these barriers.

**Trial registration:**

ISRCTN81494090, 19/09/2023.

**Supplementary Information:**

The online version contains supplementary material available at 10.1186/s40814-026-01861-3.

## Key messages

- The evidence on the effectiveness of remotely delivered group-based parenting programmes, such as Group Triple P, compared to face-to-face programmes, is inconclusive.
- Remote delivery of the Group Triple P parenting intervention was generally feasible and acceptable to both parents/caregivers and programme delivery staff: however, reduced group engagement was observed.
- It would not be feasible to include individual randomisation in a future definitive trial in this trial setting/population, but other non-randomised trial designs should be considered.

## Background

Parents play a key role in the health and wellbeing of families. Family relationships are critical to children’s social and emotional development and longer-term outcomes [1, 2]. Parents’ own health and wellbeing is important and in turn shapes the quality of parenting and family relationships [3, 4]. Supporting parents is therefore a major public health priority. Many well-designed parenting interventions have demonstrated effectiveness in strengthening parenting behaviours, improving parent–child relationships and reducing child behaviour problems and prevent poor parent mental health [5]. Furthermore, the Triple P parenting programmes have be shown to have beneficial impact on parenting practices, parenting satisfaction and efficacy, parental adjustment and parental relationship [6] A significant proportion of these interventions are delivered to groups of parents who meet face-to-face, with a practitioner (or team of practitioners) over several weeks. Learning comes from the materials delivered by practitioners and the interaction between participants as they work with this material [7].

Several challenges related to the implementation of group-based parenting interventions have been identified. A longstanding issue is participant recruitment and retention [8, 9], particularly underserved groups such as fathers [10, 11] and ethnic minority populations [12]. Practical barriers to attendance, including work commitments and travel, have been highlighted as significant [13, 14]. Psychological barriers may also arise, such as discomfort in group settings or concerns about being judged on one’s parenting [13].

The COVID pandemic necessitated a rapid transfer of group-based parenting interventions to remote delivery using online videoconferencing platforms, such as Microsoft (MS) Teams and Zoom. Post-pandemic, use of remote delivery has continued and has been integrated into the routine practice of many providers. There is growing interest in whether remote delivery might address key challenges experienced by face-to-face group-based interventions, including recruitment and retention of underserved groups, and reducing practical and psychological barriers to attendance. However, it is also important to consider how with the move to remote delivery it may be possible to avoid inadvertently generating new forms of inequality including those driven by digital poverty.

Research on the adaptation of group-based parenting interventions for remote delivery and their subsequent implementation is still limited [15, 16], but a number of studies have demonstrated feasibility and acceptability [17–19]. For example, Papakonstantinou Rodi, et al. [19] explored practitioners’ experience of remote delivery of the Stepping Stones Group Triple P intervention. Overall, remote delivery achieved good acceptability (among parents and practitioners), allowing scarce resources to be ‘stretched’ (due to lower running costs), and the opportunity for staff to gain new skills. However, a recent systematic review found that many studies reported poorly on the adaptations made for remote delivery and gave little detail on how implementation fidelity was measured or maintained [17].

One of the key potential benefits of remote delivery is that it offers participants flexibility and convenience, reducing barriers to attendance [15, 18], including removing the need to travel to meeting venues. Some studies have found that remote delivery achieves high rates of participation (compared with face-to-face delivery) [19, 20]. However, evidence on the extent to which it addresses recruitment challenges (particularly among underserved groups) is limited and mixed. Whilst McDevitt [21] found that remote delivery increased reach within remote and underserved communities, other researchers have reported broadly analogous demographic participant profiles when implemented alongside face-to-face provision of the same intervention [22, 23]. There is a paucity of evidence on whether remote delivery achieves higher participation rates among fathers and members of ethnic minority groups.

In some cases, remote delivery has encountered continued barriers to engagement, including participants’ variable access to stable internet connections and suitable digital devices [17, 20]. Another identified barrier to increasing participant diversity is meeting the needs of individuals whose primary language differs from the one used to deliver the intervention, and how best to provide real time interpretation.

The evidence on the effectiveness of remotely delivered group-based parenting programmes remains inconclusive, including in comparison with face-to-face formats [17, 24]. Papakonstantinou et al. (2024) found that practitioners perceived remote delivery as matching the effectiveness of face-to-face and believed that most participants preferred the former. A common theme across studies is that whilst practitioners were able to deliver key content and interact with participants, it was often challenging to promote group interaction and learning (which have been identified as important intervention mechanisms) [15, 19, 20]. For instance, Canário et al. [15] reported difficulties in recreating role plays by parent dyads, and Cook et al. [20] describe challenges with promoting learning between participants.

Provision of group-based parenting programmes for remote delivery therefore offers significant promise in terms of acceptability and feasibility. In addition, reach of underserved populations, including diverse ethnic groups and fathers, may improve by increasing accessibility of the intervention. However, research is still limited on the implementation of remotely delivered interventions and their effectiveness on key outcomes [15, 17, 22, 24]. There is uncertainty concerning the extent to which remote delivery addresses participant engagement and reach (and in what contexts). Few studies have explored how to manage group interaction and cohesion [15] and the implications for intervention mechanisms. The Group Triple P intervention is implemented face-to-face and remotely by Local Authorities and independent providers and is therefore a good candidate to explore the feasibility of implementing both a remotely and face-to-face delivered parenting intervention in a trial context.

## Methods

### Aim and objectives

The aim of the trial was to assess the feasibility of delivering the Group Triple P intervention to parents/caregivers of young children (0–12 years) by Local Authority or independent service provider organisations. The feasibility trial was designed to inform a potential, definitive trial of the effectiveness and cost-effectiveness of Group Triple P delivered remotely compared to face-to-face delivery.

The main objectives were to assess (1) the feasibility of recruiting suitable sites (Local Authority and independent service provider organisations); (2) the feasibility of recruiting eligible participants to the trial; (3) retention through the 16-week follow-up data collection; (4) the acceptability of trial processes, including randomisation, to site staff, practitioners and parents/caregivers; (5) acceptability of intervention delivery (including an assessment of preference of delivery methods) to site staff, practitioners and parents/caregivers; (6) fidelity of implementation of Group Triple P, reach and adherence to the intervention; (7) the feasibility and acceptability of proposed outcome measures for a definitive trial; (8) the feasibility and acceptability of collecting resource use, cost and health-related quality of life (parent and child) data, as methods for conducting an embedded health economic evaluation within a definitive trial; (9) the acceptability of collecting and analysing routine data within a definitive trial and (10) Local Authority and independent service provider willingness to participate in a definitive trial.

### Design and setting

The trial was a feasibility non-randomised trial. The trial took place in four sites; three Local Authority and one independent provider, across England and Wales. Site inclusion required that one face-to-face and one remote Group Triple P programme would be delivered simultaneously.

### Participants

Participants included parents/caregivers who: (1) were a biological, step, adoptive or foster carer of at least one child aged 0–12 years; (2) cared for the child for a substantial part of each week; (3) were aged at least 18 years; (4) spoke sufficient English to attend the intervention, consent and complete questionnaires over the telephone; and (5) had not previously attended the Group Triple P parenting programme. Participants were identified by sites, who utilised existing recruitment pathways. Name and contact details of interested participants were registered on an online survey and transferred to the research team. Parents/caregivers completed the online recruitment process via email links to the trial database; participants accessed the Participant Information Sheet, completed screening information and provided informed consent. If applicable and the child was willing, participants were also asked to provide consent for their child (at least 8 years old) to complete outcome measures. Participants and children were offered alternative telephone screening and informed consent.

### Allocation

Participants were not randomised to intervention allocation; participants were allocated to either remote or face-to-face intervention delivery via participant preference prior to consent.

### Sample size calculation

No power calculations were performed as the aim was to evaluate feasibility. A total of 4 sites were to be recruited to deliver two intervention group sessions: one face-to-face and one remotely delivered. Between 8 and 12 participants were to attend each group, resulting in a planned sample size of at least 64 participants (maximum of 96 participants). The number of completed groups will provide sufficient data to estimate parameters required for determining recruitment, response rates, intervention acceptability and adherence. For the initial randomised trial design, *n* = 64 would allow the estimation of an expected retention proportion of 0.7 to within ± 0.112 using a 95% confidence interval.

### Data collection

Participant-reported outcome measures (PROMs) were collected online via an email, linked to the trial database, sent to the participants at two timepoints—baseline and 16 weeks (see Table 1). Participants and children were offered alternative telephone data collection with researchers blinded to allocation. Any incidents of unblinding were recorded. PROMs for parent/caregivers included (1) children’s behavioural and emotional problems; (2) reduced lax or over reactive parenting; (3) positive parenting, parent–child relationships, family relationships and parental team work; (4) parental confidence; (5) parental wellbeing; (6) demographics (age, sex, gender, ethnicity, educational qualifications, occupational status, family and household composition); (7) parent health-related quality of life; (8) health economics resource use; (9) child health-related quality of life proxy; (10) participant views on randomisation in a future trial and (11) participant views on use of routinely collected data in a future trial. PROMs for children (8 years old and over) included (1) children’s behavioural and emotional problems: Me and My Feelings Questionnaire; (2) health and family relationships; and (3) child health-related quality of life. See Table 1 for details of PROMs data collection timings.

**Table 1:** Timings of PROMs

Outcome measure	Timepoints	
	**Baseline**	**16 weeks follow-up**
Participant-reported:		
Demographics: age, sex, gender, ethnicity, educational qualifications, occupational status, family and household composition	X	
Children’s behavioural and emotional problems: Strengths and Difficulties questionnaire (SDQ) [25]	X	X
Reduced lax or over reactive parenting: Arnold and O’Leary Parenting Scale (APS) [26]	X	X
Positive parenting, parent–child relationship, family relationship and parental team work: Parenting and Family Adjustment Scales (PAFAS) [27]	X	X
Parental wellbeing: Parental confidence: Parenting Sense of Competence Scale [28]	X	X
Parental wellbeing: Depression Anxiety Stress Scale (DASS) [29]	X	X
Parent health-related quality of life: EQ-5D-5L [30]	X	X
Health economics resource use: Adapted Client Service Receipt Inventory (CSRI) [31]	X	X
Child health-related quality of life: Child Health Utility 9 Dimension Instrument: CHU9D proxy [32]	X	X
Acceptability of the use of randomisation in a future definitive trial		X
Acceptability of the use of routinely collected data in a future definitive trial		X
Child (8 years and older) reported:		
Children’s behavioural and emotional problems: Me and My Feelings Questionnaire [33]	X	X
Health and family relationships: Kidscreen 27 [34]	X	X
Child health-related quality of life: Child Health Utility 9 Dimension Instrument (CHU9D) [32]	X	X
Child health-related quality of life: EQ-5D-Y [35]	X	X

### Process evaluation

Drawing on MRC guidance [36], a mixed methods process evaluation explored (1) key influences on site and participant recruitment; (2) the feasibility of delivering Group Triple P remotely with fidelity; (3) the views of practitioners on the acceptability of both trial processes (such as randomisation and online data collection including informed consent) and of remote delivery of Group Triple P; (4) the views of parents/caregivers on the acceptability of trial processes and of remote delivery of Group Triple P; (5) the views of practitioners and parents/caregivers on the acceptability and feasibility of a potential definitive trial. The following data was collected as part of the process evaluation: (1) attendance logs and practitioner-completed fidelity checklists for remote and face-to-face intervention group sessions; (2) qualitative interviews (conducted via videoconferencing) with site staff, programme delivery staff and parents/caregivers; and (3) observation of remotely delivered intervention sessions.

#### Attendance logs and practitioner-completed fidelity checklists

Practitioners were requested to complete attendance logs (developed by the research team, detailing the attendance at group sessions on a per participant basis) and fidelity checklists (developed by Triple P, detailing the delivery of session components per session) following each Group Triple P session. Forms were returned to the research team via secure file transfer.

#### Qualitative interviews (conducted via Zoom) with site staff, programme delivery staff and parents/caregivers

Programme delivery staff (up to 10) and parents/caregivers (up to 20) were invited to participate in a qualitative interview and provided with an information sheet. Parents/caregivers provided consent to be contacted to take part in a qualitative interview. Parents/caregivers were then sampled to ensure balance across sites and to include fathers. The interviews were conducted over videoconferencing at a time convenient for the participant and verbal informed consent obtained prior to the interview.

#### Observation of intervention delivery

Two sessions of Group Triple P were observed (via attendance by a research team member or by recording remote sessions) for remotely delivered groups. Group 4 and 8 group-delivered sessions were selected, rather than one-to-one sessions, and later in the programme so as not to disrupt group dynamics in earlier sessions. Observations required verbal consent from all participating attendees prior to the start of the group; attendees were provided with an information sheet and were given sufficient opportunity to anonymously express concerns to the facilitator prior to the session. Parents were able to withdraw consent at any point in the session and the observation would be stopped. Observers did not formally assess implementation fidelity, but completed an observation checklist (with questions on what was delivered, participant response and any technical or other challenges encountered).

### Economic analysis

The economic analysis aimed to assess the feasibility and acceptability of collecting resource use, cost and health-related quality of life (parent and child) data, as part of a future definitive trial. The following data were collected: (1) data on resource use, cost and outcome measures required for a health economic evaluation (adapted-CSRI and health-related quality of life measures) were collected in participant and child PROMs; (2) site/delivery staff completed a standard proforma, which detailed retrospective intervention implementation costs, including costs for set-up, programme delivery, monitoring and follow-up of clients, administrative activities, plus any miscellaneous costs; and (3) a subsequent meeting with the health economist clarified detail in the proforma and provided a more in-depth cost narrative.

### Analysis

Prior to all analyses, the following Study Steering Committee (SSC)-approved analysis plans were developed: (1) a statistical and health economics analysis plan (SHEAP), developed by the trial statistician and health economist and (2) a qualitative analysis plan (QAP), developed by the trial qualitative researcher.

#### Statistical analysis

The trial protocol followed SPIRIT (Standard Protocol Items: Recommendations for Interventional Trials) guidelines and due to the inclusion of randomisation in the initial trial design, analysis was conducted in accordance with CONSORT (Consolidated Standards of Reporting Trials) guidelines. Significance tests were not reported as the trial was not powered to test hypotheses. The majority of outcome analysis was descriptive and assessed against pre-determined criteria to inform continuation to a definitive trial. Baseline demographic/outcome scores were summarised and tabulated by arm. Estimates of the variance of potential primary/secondary outcomes for a future definitive trial were examined. Patterns in attendance, withdrawal and loss to follow-up and drop out were reviewed.

#### Process evaluation analysis

Thematic analysis [37] was used to analyse each group of interviews separately and independently followed by qualitative synthesis across all interviews to provide an over-arching synthesis of participants’ views on intervention feasibility, acceptability and mechanism. Intervention attendance and adherence was explored in both arms and fidelity checklists were subjected to descriptive analysis. A triangulation exercise was conducted combining qualitative and quantitative data analysis results, mapped on to the study objectives.

#### Economic analysis

The trial protocol followed Consolidated Health Economic Evaluation Reporting Standards (CHEERS) 2022 [38] reporting guidelines. The methodological approach included (1) an early assessment of the economic costs associated with delivering Group Triple P remotely or face-to-face; (2) an assessment of the broader resource use and health-related quality outcomes associated with delivering Group Triple P remotely or face-to-face; (3) identification of appropriate sources of unit costs for potential resource consequences and an assessment of how much primary costing research would be required for the main trial; (4) identification of available routine health and social data sources that could be used to complement and validate self-reported resource utilisation data; and (5) an assessment of the best approach for expressing cost-effectiveness in a future trial.

### Intervention

The Triple P (Positive Parenting Programme) is a system of five levels of evidence-based, parenting and family support interventions. Level 4 Group Triple P is a group parenting programme for parents/caregivers of children up to the age of 12 [39]. Parents/caregivers attend the intervention in groups of up to 12 parents/caregivers, over 8 weeks, to develop strategies to build healthy relationships and confidently manage their children’s behaviour. The intervention consists of five (2 h) group sessions and three (15–30 min) individual telephone consultations. Practitioners are trained by and implementation supported by Triple P UK (TPUK). In group sessions, parents/caregivers learn from one another and participate in activities to learn about the causes of child behaviour problems, setting specific goals, and using strategies to promote child development and manage misbehaviour. Individual telephone consultations encourage parents/caregivers’ independent problem-solving following implementing the learnt skills at home.

### Changes to the initial protocol

Two significant changes to the protocol were approved by the Study Steering Committee and the Cardiff University School of Medicine Ethics Committee. These included (1) following feedback from sites, participant randomisation was removed for the trial design and replaced by participant-selection of intervention allocation, and (2) the inclusion of independent providers, as well as Local Authority sites.

### Public involvement

Members of the public contributed to the development of the study proposal, including inputting in the value of the research, the selection of aims/objectives, and development of the study lay summary. Two public involvement groups were consulted: (1) public members of DECIPHer Centre’s young people’s advisory group ALPHA and (2) parents in an ethnically diverse part of Cardiff, facilitated by a Cardiff University Community Engagement project. Public involvement in the trial included (1) Study Steering Committee (SSC) contribution by a parent; (2) Contribution to the trial materials, via face-to-face meetings, by a Parent’s Advisory Group (PAG); (3) Contribution by DECIPHer Centre’s young people’s advisory group ALPHA informed the study findings and advised on dissemination of key results to participants.

## Results

### The feasibility of recruiting suitable sites (Local Authority and independent service provider organisations)

Four sites, including three Local Authority sites and one independent provider of Group Triple P across England and Wales, were recruited, as per protocol. However, site recruitment was challenging for several reasons: (1) accurate information regarding the number and location of sites delivering group Triple P was lacking, which made it challenging identifying potential sites; (2) resourcing of facilitators to deliver two concurrent Group Triple P programmes was problematic at many sites; (3) the number of research-active sites was minimal and reduced further by a large research study delivered simultaneously with the 3P Study and (4) some sites were ineligible due to strategic decisions to offer only one delivery method of Group Triple P.

### The feasibility of recruiting eligible participants to the trial

**Fig. 1:**
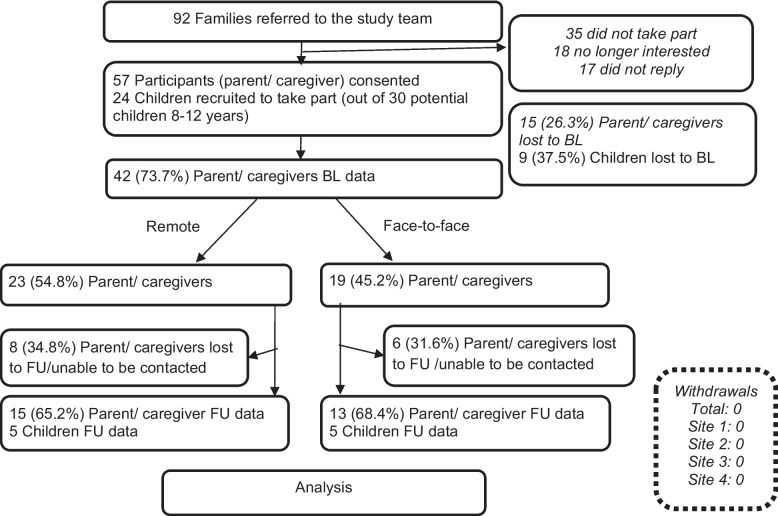
CONSORT diagram

Individual sites were required to refer parents/caregivers in order to recruit at least 16 participants (a minimum of 8 parents per Group Triple P group). Out of a total of 92 parents/caregivers who expressed interest to the research team, 57 participants provided online informed consent, representing 89.1% of the recruitment target and a 62% recruitment rate (Table 2). However, total parent referral and participant consent rates varied across sites, ranging between 15 − 32 parents/caregivers and 7 − 19 participants (see Table 2 for participant referral and recruitment rates per site). Out of the total consented participants, 42 (73.7%) completed baseline data collection with 15 participants lost to the baseline data collection timepoint. Thirty participants reported that their target child was 8 years old or older and 24 participants (80%) consented to their child completing trial data. A total of 15 children (62.5%) provided baseline data. For the purposes of reporting, consented parents who completed baseline data were considered trial participants (*n* = 42). See Fig. 1 for the CONSORT diagram.

**Table 2:** Participant referral and recruitment rates per site

	Site 1	Site 2	Site 3	Site 4	Total
Number of parents/caregivers referred to the research team	15	32	23	22	92
Number of consented parents/caregivers	7	19	16	15	57
Number of trial participants (consented and completed baseline data)	5	9	15	13	42

Recruitment processes varied between sites, depending on their existing pathways for identification of suitable families and the site setting e.g. Local Authority versus independent providers. In Local Authority sites, participants were referred formally via various professionals including social workers, health visitors, GPs, school engagement teams, and family intervention services. All sites advertised the trial using posters and leaflets with details of a parent/caregiver self-referral process, in various settings including (1) social media including Facebook, Instagram and X; (2) Youtube; (3) school newsletters and premises; (4) GP surgeries, health and social care professionals; and (5) other community settings. Sites also provided trial information directly to parents/caregivers who were registered on pre-existing contact lists. Following initial referral/self-referral of parents/caregivers, many sites used interview or screening processes to establish the family’s unique support needs, including geographic location or preference for intervention delivery method, and to determine whether Group Triple P was the best fit. Central systems/record management systems were also used in most sites to streamline identification/recruitment of parents/caregivers.

Barriers to effective identification of potential participants differed depending on the site setting i.e. Local Authority versus independent provider sites. A lack of referral pathways or the potential to form established links with health visitors or educational settings was a barrier to the independent provider site, resulting in a greater dependence on recruitment pathways using social media and community newsletters. Conversely, Local Authority sites successfully utilised existing links with healthcare professionals, social care professionals and schools to identify potential families. Other facilitators to participant recruitment included (1) using a formalised process to allow referral from various professions; and (2) undertaking a screening process whereby specialists assessed the specific requirements of families (including challenges that need addressing, family preferences for location, timing and method of delivery of sessions) in order to identify the most appropriate programme available, which may not be Group Triple P. As face-to-face and remote sessions were delivered concurrently, the same recruitment pathways were utilised for both programmes.

We get referrals from social care, from intervention workers, from schools, from GPs, and that all comes into one tray. Once we know they’re in the zero to twelve age bracket, we assess which programme would best suit the family. [Site staff, Site 2]

A parenting specialist will discuss the referral with the family to determine the most appropriate programme. Sometimes the concerns might align better with a team-based programme, or there may be a child with additional needs that would make a different programme more appropriate. [Site staff Site 2]

Principally, usually I go straight to the schools, because that makes sense. But the issue I have with schools is they don’t know me. So I am just another email that pops in. [Site staff, Site 3]

Participants’ baseline demographic data is provided in Table 3. The majority of participants were married, white (92.8%), female (92.9%), aged 30–39 (45.2%), spoke English as a first language (85.4%) and were the mother of the child who was the focus of the Triple P intervention (92.9%). A substantial proportion of the participants had a longstanding illness, disability or infirmity (33.3%) and reported fair or bad health (35.7%). There were no large differences between those who attended the programme face-to-face compared to those who attended remotely.

**Table 3:** Baseline demographic summary data

Variable	Baseline (*n* = 42)	Remote (*n* = 23)	Face-to-face (*n* = 19)
**Age of parent**			
Less than 30	9 (21.4%)	4 (17.4%)	5 (26.3%)
30 to 39	19 (45.2%)	11 (47.8%)	8 (42.1%)
40 and over	14 (33.3%)	8 (34.8%)	6 (31.6%)
**What gender were you assigned at birth?**			
Male	3 (7.1%)	2 (8.7%)	1 (5.3%)
Female	39 (92.9%)	21 (91.3%)	18 (94.7)
**What gender do you identify as?**			
Male	2 (4.8%)	2 (8.7%)	0 (0%)
Female	36 (85.7%)	20 (87.0%)	16 (84.2)
Non-binary	1 (2.4%)	0 (0%)	1 (5.3%)
Would rather not answer	3 (7.1%)	1 (4.3%)	2 (10.5%)
**Is English your first language?**			
No	6 (14.6%)	4 (18.2%)	2 (10.5%)
Yes	35 (85.4%)	18 (81.8%)	17 (89.5%)
*missing*	*n* = *1*	*n* = *1*	
**Ethnicity**			
White—British	31 (73.8%)	17 (73.9%)	14 (73.7%)
White—Other	8 (19.0%)	5 (21.7%)	3 (15.8%)
Asian or Asian British—Indian or Pakistani, Black or Black British—Caribbean	3 (7.1%)	1 (4.3%)	2 (10.5%)
**What is your marital or civil partnership status?**			
Single	11 (26.2%)	7 (30.4%)	4 (21.1%)
Married and living with spouse/civil partner	17 (40.5%)	7 (30.4%)	10 (52.6%)
Divorced/separated	5 (11.9%)	2 (8.7%)	3 (15.8%)
Living with partner, but not married or in a civil partnership	8 (19.0%)	7 (30.4%)	1 (5.3%)
Not currently living with partner	1 (2.4%)	0 (0%)	1 (5.3%)
**How is your health in general?**			
Very good	9 (21.4%)	5 (21.7%)	4 (21.1%)
Good	18 (42.9%)	9 (39.1%)	9 (47.4%)
Fair	14 (33.3%)	8 (34.8%)	6 (31.6%)
Bad	1 (2.4%)	1 (4.3%)	0 (0%)
**Do you have a longstanding illness, disability or infirmity?**			
No	28 (66.7%)	17 (73.9%)	11 (57.9%)
Yes	14 (33.3%)	6 (26.1%)	8 (42.1%)
**What relationship are you to the child who is being focused on in Triple P?**			
Mother	39 (92.9%)	21 (91.3%)	18 (94.7%)
Father	2 (4.8%)	2 (8.7%)	0 (0%)
Other	1 (2.4%)	0 (0%)	1 (5.3%)

Summary data for educational attainment, employment and household finances are provided in Table 4. The majority were employed in professional occupations (69.6%) with varied levels of qualifications and household income. Most data is comparable between trial arms, with the exception of education; participants in the remote arm were qualified to a higher level compared to participants in the face-to-face arm.

**Table 4:** Baseline education, employment and finance data

Variable	Baseline (*n* = 42)	Remote (*n* = 23)	Face-to-face (*n* = 19)
**What is your highest level of educational qualification?**			
No academic or professional qualifications	4 (19.5%)	0 (0%)	4 (21.1%)
GCSEs or equivalent: O Levels/CSE, Entry Level, Foundation Diploma, NVQ level 1, 1 A Level/2–3 AS, Apprenticeship or Vocational/Work-related Qualifications, Foreign Qualifications	16 (38.1%)	8 (34.8%)	8 (42.1%)
2 + A Levels or equivalent (Level 3 qualifications): 2 + A Levels/VCEs, 4 + AS Levels, Higher School Certificate	6 (14.3%)	3 (13.0%)	3 (15.8%)
Degree level (Level 4 qualifications and above) or Higher Degree	16 (38.1%)	12 (52.2%)	4 (21.1%)
**What is your current employment status?**			
Employed/self-employed	25 (59.5%)	15 (65.2%)	10 (52.6%)
Unable to work, Homemaker, Student, out of work	17 (40.5%)	8 (34.8%)	9 (47.4%)
**What describes your current occupation best?**			
Professional: business, healthcare, technology, science	16 (69.6%)	11 (78.6%)	5 (55.6%)
Skilled and unskilled trades, office, caring, operatives and manufacturing occupations	7 (30.4%)	3 (21.4%)	4 (44.4%)
*Missing/not applicable*	*n* = *19*	*n* = *9*	*n* = *10*
**Have you lost days of work due to your or your child’s health?**			
No	32 (78.0%)	18 (81.8%)	14 (73.7%)
Yes	9 (22.0%)	4 (18.2%)	5 (26.3%)
*missing*	*n* = *1*	*n* = *1*	
**Have you incurred any additional expenses, e.g. childcare costs over the last 16 weeks?**			
No	34 (81.0%)	18 (78.3%)	16 (84.2%)
Yes	8 (19.0%)	5 (21.7%)	3 (15.8%)
**What is your total weekly household income (after any deductions, e.g. income tax)**			
£0–£500	12 (28.6%)	6 (26.1%)	6 (31.6%)
£501–1000	14 (33.3%)	7 (34.8%)	7 (36.8%)
£1000 +	2 (4.8%)	2 (8.7%)	0 (0%)
Prefer not to say	14 (33.3%)	8 (34.8%)	6 (31.6%)
**How well would you say you [and your husband/wife/partner] are managing financially these days?**			
Living comfortably?	11 (26.8%)	6 (26.1%)	5 (27.8%)
Doing alright?	14 (34.1%)	8 (34.8%)	6 (33.3%)
Just about getting by?	14 (34.1%)	9 (39.1%)	5 (27.8%)
Finding it quite difficult?	2 (4.9%)	0 (0%)	2 (11.1%)
*Missing*	*n* = *1*		*n* = *1*
**Suppose you only had 1 week to raise £2000 for an emergency, which of the following best describes how hard it would be for you to get that money?**			
I could easily raise the money	13 (31.0%)	7 (30.4%)	6 (31.6%)
I could raise the money, but it would involve some sacrifices (e.g. reduced spending, selling a possession)	8 (19.0%)	6 (26.1%)	2 (10.5%)
I would have to do something drastic to raise the money (e.g. selling an important possession)	5 (11.9%)	4 (17.4%)	1 (5.3%)
I don’t think I could raise the money	16 (38.1%)	6 (26.1%)	10 (52.6%)

Household composition data are summarised in Table 5. The majority of households comprised two adults (58.5%) and two children (50.0%), with the second adult in most cases being the other parent (80.6%).

**Table 5:** Household composition

Variable	Baseline (*n* = 42)	Remote (*n* = 23)	Face-to-face (*n* = 19)
**How many adults live in your household (excluding yourself)?**			
0	11 (26.8%)	5 (22.7%)	6 (31.6%)
1	24 (58.5%)	14 (63.6%)	10 (52.6%)
2	3 (7.3%)	1 (4.5%)	2 (10.5%)
3	2 (4.9%)	1 (4.5%)	1 (5.3%)
4	1 (2.4%)	1 (4.5%)	0 (0.0%)
*missing*	*n* = *1*	*n* = *1*	
**What relation are they to the child who is being focused on during Triple P?**			
Mother	5 (16.1%)	2 (11.8%)	3 (21.4%)
Father	20 (64.5%)	10 (58.8%)	10 (71.4%)
Stepdad	2 (6.5%)	2 (11.8%)	0 (0.0%)
Adult Sister	1 (3.2%)	1 (4.3%)	0 (0.0%)
Grandmother	1 (3.2%)	1 (4.3%)	0 (0.0%)
Grandfather	1 (3.2%)	1 (4.3%)	0 (0.0%)
No relation	1 (3.2%)	0 (0%)	1 (7.1%)
*Missing/not applicable*	*n* = *11*	*n* = *6*	*n* = *5*
**How many children live in your household?**			
1	12 (28.6%)	5 (21.7%)	7 (36.8%)
2	21 (50.0%)	13 (56.5%)	8 (42.1%)
3	4 (9.5%)	2 (8.7%)	2 (10.5%)
4	2 (4.8%)	2 (8.7%)	0 (0.0%)
5	2 (4.8%)	1 (4.3%)	1 (5.3%)
8	1 (2.4%)	0 (0.0%)	1 (5.3%)

Table 6 describes the baseline demographics for the nominated child who was the focus of the Triple P programme. Most nominated children were in mainstream primary school (88.1%), with 19.0% of children reported as having a disability.

**Table 6:** Nominated child baseline demographics

Variable	Baseline (*n* = 42)	Remote (*n* = 23)	Face-to-face (*n* = 19)
**What type of school does the nominated child attend?**			
Not in school	3 (7.1%)	1 (4.3%)	2 (10.5%)
Mainstream primary school	37 (88.1%)	22 (95.7%)	15 (78.9%)
Mainstream primary school with special educational needs	1 (2.4%)	0 (0.0%)	1 (5.3%)
Special school	1 (2.4%)	0 (0.0%)	1 (5.3%)
**Does the nominated child have a disability?**			
No	34 (81.0%)	20 (87.0%)	14 (73.7%)
Yes	8 (19.0%)	3 (13.0%)	5 (26.3%)

### Participant retention at the 16-week follow-up data collection

Data collection rates at the 16-week follow-up timepoint are presented in the CONSORT diagram (1). At the 16-week follow-up, 62.5% (*n* = 15) of participants in the remote trial arm and 72.2% (*n* = 13) of participants in the face-to-face trial arm completed data collection. Child data was completed for five parents/caregivers in the remote delivery arm and for five parents/caregivers in the face-to-face trial arm. Participants were contacted up to 3 times via email with a link to their follow-up data collection forms and were reminded by site staff at routine contacts.

### The acceptability of trial processes, including randomisation, to site staff, practitioners and parents/caregivers

The inclusion of randomisation to 2 trial arms (one arm attending remotely delivered Group Triple P and one arm attending face-to-face delivered Group Triple P) proved to be a significant barrier to both site and participant recruitment. Several Local Authorities were unable to participate in a trial including a randomisation element for several reasons: (1) strategic decisions meaning that randomisation and running concurrent group programmes were not possible; (2) the site staff expressed concern that the inclusion of randomisation would result in the removal of parent/caregiver choice, which is essential to accommodate families’ needs and schedules and (3) site staff expressed that by allowing families the flexibility to select the delivery method that best suited their personal and family circumstances made the programme more accessible and removal of that flexibility would result in families unable to take part in the trial. Similarly, parents/caregivers described that the option to choose delivery method was a crucial factor in their decision to engage with the programme and that randomisation would be a challenge to trial participation. When questioned whether the inclusion of randomisation in a future definitive trial would change the likelihood of whether or not they would take part, 50% of participants stated that randomisation would make no difference to the likelihood of them taking part in a trial, but 37.5% of participants said that they would be less likely to take part in a trial involving randomisation (see Table 7).

**Table 7:** Participant/caregivers’ views surrounding incorporation of randomisation in a future definitive trial

	Parents/caregivers
	(***n*** = 32)
The impact of including randomisation to attend face-to-face or remote (via Teams/Zoom and Whatsapp) Group Triple P sessions in a future definitive trial	***n*** (%)
Definitely less likely to take part	5 (15.6)
Slightly less likely to take part	7 (21.9)
No difference	16 (50.0)
Slightly more likely to take part	3 (9.4)
Definitely more likely to take part	1 (3.1)
Missing	0 (0)

At the time, it was between whether it would be randomised, whether it would be online, or in person, and I think that would have affected it a little bit, because the dynamics of how I look after the kids, when my partner’s [away], meant that going out would be a bit more challenging. So, um, anyway, as it turns out, it was online, which was great, so I could, I could be here and um, listen and participate. [Participant, Site 3]

[Randomisation] was a bit of a nightmare. People like to plan, right, so I couldn’t tell them whether they’d got the day they needed, until I got enough people. Then you get enough people, and they say, ‘Oh no, I can’t now, because I’ve booked a yoga class or something’. [Site staff, Site 3]

If we would have randomised it and just told them, ‘You’re going to face-to-face or you’re going to online,’ they wouldn’t have come, no way. There’s still quite a stigma or resistance to parenting groups. [Site staff, Site 4]

**Fig. 2:**
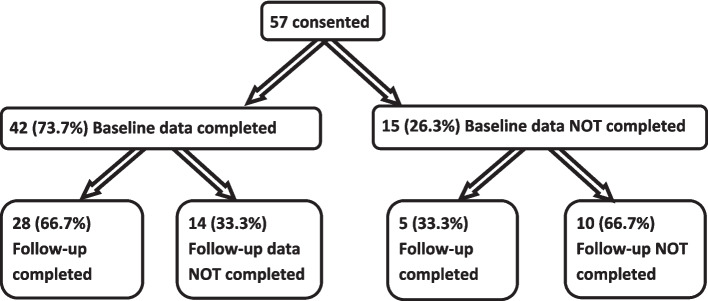
Consented parent/caregiver data collection flow diagram

Participant self-selection to either trial arm (remote versus face-to-face programme delivery) replaced the randomisation element of the trial design. Of the 42 trial recruits, 23 participants self-selected to attend the remote delivery of Group Triple P programme and 19 participants self-selected to attend the face-to-face Group Triple P programme. A consequence of this trial design was that sites were not dependent on the trial team to inform them of the participant’s allocation to trial arm, resulting in site staff arranging attendance at intervention sessions when participants had not completed all recruitment stages (e.g. consent, baseline data completion). A total of 15 (26.3%) consented parents/caregivers did not complete baseline data collection, some of whom subsequently completed follow-up data collection (see Fig. 2). Only participants who completed baseline data collection were reported in this trial.

### Acceptability of intervention delivery to site staff, practitioners and parents/caregivers

The option to choose remote delivery was a crucial factor in some parent/caregivers’ decision to engage with the trial and Group Triple P programme. Logistical barriers to attending face-to-face intervention sessions included work schedules, travel requirements, childcare responsibilities and the absence of family support. In addition, some participants described anxiety of being in social situations. These barriers were mitigated and accessibility to the programme improved by offering remote delivery. Furthermore, for some families, remote delivery facilitated both parents/caregivers attending the programme together, an important consideration for some families. Alternatively, some parents expressed a preference for face-to-face sessions, considering that this format may provide a richer, more interactive experience.

My partner is also a doctor and with shift work and on-calls, you know, there’s no consistency, so on that evening, I could always get there... I don’t have to worry about getting into [City], trying to park, you know, that was gonna be an extra, half hour, three quarters of hour, which I couldn’t account for. [Participant, Site 3].

But online for a lot of parents is really helpful, especially if they’ve got anxieties about coming into a strange building, getting on and off buses, different car journeys. Time is a major issue. [Participant, Site 2]

I’m just not one for sitting in a room with people I don’t know. [Participant, Site 3]

The online meant that we could both take part and both be involved in the learning... I don’t think it would have been as valuable if I had been coming home from an in-person course and reported back. [Participant, Site 3]

Obviously, in an ideal world, I’d do it in person... I would love for me and [name] to go into a room and do it together, a hundred percent, but that is not our life... our experience would have been completely different. [Participant, Site 3]

Participant engagement was hampered by remote delivery; site staff reported both lack of interaction between participants or the facilitators and difficulties in eliciting meaningful feedback from parents during their online sessions. Site staff reported that the use of cameras was inconsistent, meaning it was difficult to determine whether participants understood the course content. Furthermore, lack of visual interaction hindered meaningful relationship building, peer support and collaborative learning between participants—elements that are central to the Triple P programme’s approach. Observations of remote group sessions also highlighted a lack of group interaction; some participants did not use their camera at all, there was little interaction between parents/caregivers and some participants did not speak at all or communicated exclusively via the videoconferencing chat message function. Participants also noted that during remote delivery, the absence of informal social interactions that are likely to occur during face-to-face group delivery meant that they were less likely to feel comfortable enough to expose vulnerabilities and share personal experiences with other parents/caregivers. Site staff noted that this was exacerbated in groups with few parents/caregivers. However, in some sites the use of breakout rooms (an effective tool within online videoconferencing software) facilitated meaningful exchange and social interaction among participants attending the remote sessions. Researchers also observed parents exchanging information and directly drawing on others’ experiences in breakout rooms. There was, however, some reluctance from facilitators to use breakout rooms if the group participants were particularly disengaged.

One practitioner successfully utilised additional digital tools to replicate some of the interactive elements typically used in face-to-face settings, including an interactive flipchart, allowing participants to contribute ideas, immediately visible to other members of the group. This approach was integrated well in the session and particularly well-received by participants.

Having been someone who’s always worked face-to-face, you know, with the pandemic, everything was put virtual... There’s not that informal conversation, that sort of soft discussion that can go alongside. And not knowing any of these individuals, you do feel a little bit more spotlighted... you feel vulnerable and exposed, because actually, it’s your life and you’re trying your best, so it’s harder to share. [Participant, Site 3]

… if there’s a couple of people, or a number of people without the cameras on, then it’s, it’s harder to build that group cohesion and group connection, people won’t share as much … [Site staff, Site 2]

Yeah, so we didn’t get, whereas when there is a bigger group with parents and they’re all kind of chipping in, that kind of friendships building up and support mechanisms um that you hope parents are going to get from a programme. We didn’t get that, but what I feel that we did get is parents being able to improve them things for their-selves and the children, which is obviously the aim. [Site staff, Site 1]

The social cues that you just don’t pick up on … you can’t do that here. And if … if I sense that somebody’s just taken an intake of breath, or they’re just looking like they want to say something, I can act on that. I can’t do that online. [Site staff, Site 3]

People are a bit … a bit quiet and stilted in the first half an hour, but they warm up once you’ve given them a chance to chat. But in the small groups there’s breakout rooms, that is the key, it really is. [Site staff, Site 3]

Course content itself was well-received by parents/caregivers. Participants described how they applied the learning from the course in their daily lives and the positive impact it had on their family life.

### Fidelity of implementation of Group Triple P, reach and adherence to the intervention

**Fig. 3:**
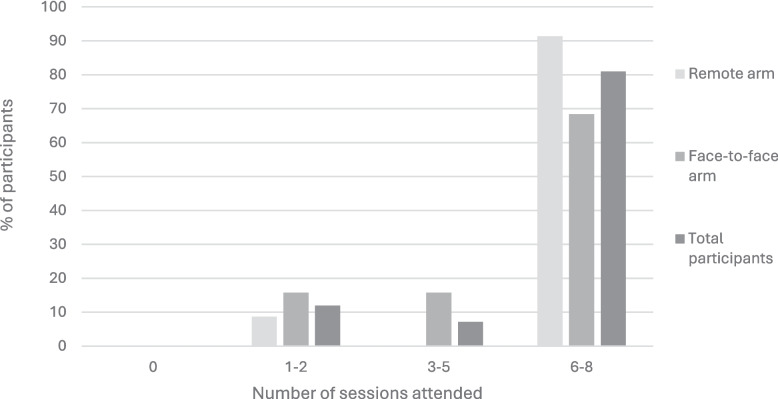
The number of sessions attended by trial participants

Most families adhered to the intervention (*n* = 27, 64.3%), defined as attending sessions 1 to 4. Whilst participants attended between 1 and 8 sessions, participants attended on average 6.3 sessions (6.6 for participants in the remote arm and 5.9 for participants in the face-to-face arm) and the majority of trial participants attended at least 6 out of the total 8 sessions (*n* = 34, 81.0%) (see Fig. 3).

**Fig. 4:**
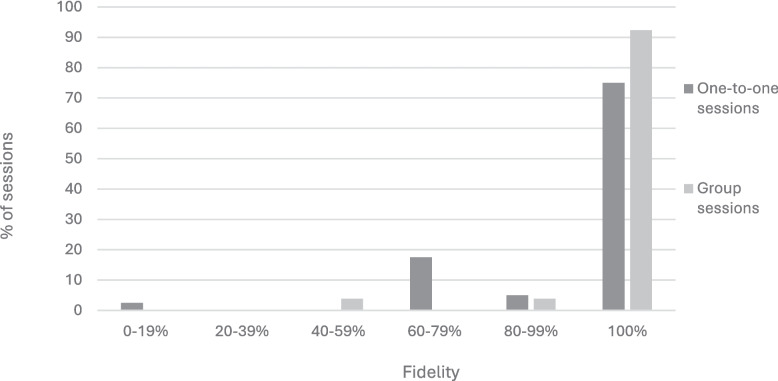
Percentage fidelity rated for group and one-to-one sessions

Fidelity to the intervention was assessed using facilitator-completed session content checklists, researcher observations of sessions and qualitative feedback from facilitators. Group Triple P session design includes 5 sessions delivered in a group format (sessions 1–4 and 8) and 3 sessions delivered as one-to-one telephone sessions (sessions 5–7). Fidelity checklists were returned by sites for 26 (72.2%) of planned group sessions and 36 (39.6%) of planned one-to-one sessions. Missing data was a result of one site not returning any fidelity checklists and deviations from intervention design; two sites delivered session 8 as a one-to-one session delivered over the telephone, rather than as a group session. Facilitator-reported fidelity to the intervention (defined as the proportion of activities that were covered in the sessions) was high. For group sessions, 92.3% of sessions were delivered with 100% fidelity and for one-to-one sessions 75.0% of sessions were delivered to 100% fidelity (see Fig. 4).

Researcher observations of remotely delivered sessions also provided evidence that sessions covered key content in the intended order and facilitators conveyed the core messages within activities. Facilitators did however reflect that several adaptations were made to facilitate session delivery and to better meet the needs of the participants. Firstly, group sessions were extended for the following reasons: (1) facilitators felt that there was insufficient time to comprehensively cover all course material and that the pace of the programme posed challenges for some parents/caregivers and (2) extra time was required to allow valued peer-to-peer discussion among parents/caregivers. Parents also reported that they felt the pace was overwhelming, with insufficient time to process and explore the content fully. Secondly, attendance at the final group session (session 8) following the three telephone calls (sessions 5–7) reduced, ultimately leading to one site optimising the intervention structure to six sessions followed by two phone calls, which proved more effective. Lastly, some sites changed how some of the activities were implemented during remote delivery; practitioners, rather than participants acted out scenarios in some role play activities and partnered activities were delivered as a group instead of in participant pairs in some groups.

The online one I ended up adding a … an extra hour to that special session, because there’s no way, I just couldn’t get through it all, um, and I was really hammering through it as well, and there’s just no way. [Site staff, Site 3]

Yeah, session six is a call. Session seven is a call and then session eight they’re meant to come back, but that’s what they found, is that they weren’t… [Site staff, Site 1]

I think we quickly, kind of, figured out that it wasn’t working, um, so, then, you know, a lot of our sessions, then were kind of, more group orientated, and then they were all kind of, talking then. [Site staff, Site 4]

Reach of the intervention to fathers was assessed. Only 2 (4.8%) of trial participants were fathers. However, a further 47.6% (*n* = 20) of participants reported that they co-habited with the father of the focus child, despite there being a significant proportion of missing data for this data point.

### The feasibility and acceptability of proposed outcome measures as methods for measuring the effectiveness of the intervention within a definitive trial

Five key participant reported outcome measures (PROMS) were completed at baseline and follow-up; these included: (1) The Strengths and Difficulties questionnaire (SDQ); (2) Arnold and O’Leary Parenting Scale (APS); (3) Parenting and Family Adjustment scales (PAFAS); (4) Parenting Sense of Competence scale; and (5) Depression, Anxiety and Stress Scale (DASS). See Tables 8, 9, 10, 11, and 12 for baseline completion numbers and scores by arms. Scores between arms were fairly similar for all measures. See Table 13 for outcome measure completion rates at baseline and follow-up. With the exception of the Parental Teamwork item on the Family Adjustment scale, completion rates were over 90% for all measures at baseline and over 80% for all measures at follow-up.

**Table 8:** Strengths and Difficulties questionnaire (SDQ) baseline scores

**Scale**	**Total**			**Remote**		**Face-to-face**
	***N***	**Mean (SD)**	***n***	**Mean (SD)**	***n***	**Mean (SD)**
Emotional Problems Scale	41	4.2 (2.4)	22	4.5 (2.4)	19	4.0 (2.4)
Conduct Problems Scale	42	4.3 (2.4)	23	4.5 (2.4)	19	4.1 (2.4)
Hyperactivity Scale	40	6.	22	7.0 (2.8)	19	6.4 (3.2)
Peer Problems Scale	42	2.9 (1.7)	23	2.7 (1.9)	19	3.1 (1.6)
Prosocial Scale	42	5.9 (2.6)	23	6.2 (2.9)	18	5.4 (2.2)
Total Difficulties Score	40	18.1 (7.7)	22	18.6 (7.7)	18	17.4 (8.0)

**Table 9:** Arnold and O’Leary Parenting Scale baseline scores

		**Total**		**Remote**		**Face-to-face**
	***N***	**Mean (SD)**	***n***	**Mean (SD)**	***n***	**Mean (SD)**
**Laxness**						
Sum	42	14.9 (5.7)	23	14.2 (6.0)	19	15.7 (5.5)
Factor Score	42	3.0 (1.1)	23	2.9 (1.2)	19	3.1 (1.1)
**Over-Reactivity**						
Sum	41	16.8 (7.0)	23	16.5 (7.2)	18	17.3 (6.9)
Factor Score	41	3.4 (1.4)	23	3.3 (1.4)	18	3.5 (1.4)
**Hostility**						
Sum	42	5.8 (3.6)	23	5.7 (3.9)	19	5.8 (3.5)
Factor Score	42	1.9 (1.2)	23	1.0 (1.3)	19	1.9 (1.2)
**No Factor**						
Sum	39	60.5 (14.9)	21	59.1 (16.2)	18	62.1 (13.6)
**Total**						
Sum	38	97.3 (27.4)	21	95.6 (30.6)	17	99.5 (23.6)
Scale Score	38	3.2 (0.9)	21	3.2 (1.0)	17	3.3 (0.8)

**Table 10:** Parenting and Family Adjustment scales (PAFAS) baseline scores

		**Total**		**Remote**		**Face-to-face**
	***N***	**Mean (SD)**	***n***	**Mean (SD)**	**n**	**Mean (SD)**
**PAFAS Parenting**						
Parental Consistency	42	6.1 (2.4)	23	5.7 (2.5)	19	6.5 (2.3)
Coercive Parenting	41	4.9 (3.2)	23	4.7 (3.0)	19	5.1 (1.2)
Positive Encouragement	42	1.6 (1.6)	23	2.1 (1.7)	19	1.0 (1.2)
Parent–Child Relationship	42	1.9 (2.2)	23	2.3 (2.6)	19	1.5 (1.5)
**PAFAS Family Adjustment**						
Parental Adjustment	42	6.2 (2.8)	23	6.1 (2.9)	19	6.3 (2.9)
Family Relationships	42	3.1 (2.2)	23	3.3 (2.4)	19	2.9 (1.9)
Parental Teamwork	33	2.5 (2.2)	18	3.1 (2.6)	15	1.9 (1.5)

**Table 11:** Parenting Sense of Competence baseline scores

		**Total**		**Remote**		**Face-to-face**
	***N***	**Mean (SD)**	***n***	**Mean (SD)**	***n***	**Mean (SD)**
Parental Satisfaction	40	22.2 (4.9)	22	22.5 (4.5)	18	21.9 (5.4)
Parental Self-Efficacy	39	35.1 (6.4)	22	36.0 (6.0)	17	34.1 (6.8)
Total	38	61.7 (10.1)	21	62.1 (8.4)	17	61.1 (12.7)

**Table 12:** Depression, Anxiety and Stress Scale (DASS) baseline scores

		**Total**		**Remote**		**Face-to-face**
	***N***	**Mean (SD)**	***n***	**Mean (SD)**	***n***	**Mean (SD)**
Depression Score	39	14.8 (13.5)	21	14.7 (14.2)	18	14.8 (13.0)
Anxiety Score	41	16.1 (14.2)	22	16.1 (14.7)	19	16.1 (13.9)
Stress Score	39	11.0 (12.5)	21	10.4 (12.9)	18	11.7 (12.4)

**Table 13:** Follow-up data collection completion rates

	Baseline			Follow-up		
	Total (***n*** = 42)	Remote (***n*** = 23)	Face-to-face (***n*** = 19)	Total (***n*** = 28)	Remote (***n*** = 15)	Face-to-face (***n*** = 13)
Strength and Difficulties Questionnaire	40 (95.2%)	22 (95.7%)	18 (94.7%)	25 (89.3%)	12 (80.0%)	13 (100%)
Parenting Scale	38 (90.5%)	21 (91.3%)	17 (89.5%)	24 (85.7%)	13 (86.7%)	11 (84.6%)
Parenting and family adjustment scale	33 (78.6%)	18 (78.2%)	15 (78.9%)	21 (75.0%)	12 (80.0%)	9 (69.2%)
Parenting sense of competence	38 (90.5%)	21 (91.3%)	17 (89.5%)	26 (92.9%)	14 (93.3%)	12 (92.3%)

Participants described several barriers to completing the trial data: (1) the length of the questionnaire and time commitment required was off-putting to participants, (2) some participants faced technical difficulties and described being unable to save their progress and return to the online questionnaire, resulting in incomplete submissions and (3) participants described uncertainty regarding which child should complete the trial questionnaire. Site staff and facilitators contacted participants by telephone or at the group sessions respectively to encourage trial questionnaire completion. Facilitators described that despite reminding participants about data collection, this did not result in completion, particularly in the remotely delivered sessions. Site staff reported that the financial incentives were a key motivating factor to encourage data completion.

I don’t remember seeing anything that was like, this survey will take up to forty minutes or something (chuckling). I remember it was like ‘holy moly, this is long’. [Participant, Site 3]

I went for my elder daughter, and then it [the programme] ended up being more for my younger daughter really, so I wasn’t sure who to get to do it. [Participant, Site 3]

### The feasibility and acceptability of methods for conducting an embedded health economic evaluation within a definitive trial

#### An early assessment of the economic costs associated with delivering Group Triple P remotely or face-to-face

The costing approach utilised an opportunity cost perspective, and all costs pertaining to the recruitment, delivery and follow-up of Group Triple P were costed irrespective of whether they accrued directly to the site or were accounted for in a different way. All sites were experienced in delivering Group Triple P and therefore design and associated planning for the intervention incurred no costs.

Costs associated with participant recruitment tasks did not differ for the remote and face-to-face delivered groups across the sites, as participant recruitment was undertaken at the same time for both groups and prior to participant arm self-selection. However, total costs incurred for participant recruitment varied from £751.68 to £1541.14 across sites. These costs were associated with various tasks including costs for promotional materials and tasks (some of which required outsourcing to neighbouring Local Authorities such as parent/caregiver screening (ranging from 1 h at one site to 2 days at another site), and family engagement prior to intervention attendance (ranging from 3 h to 4 days).

Remotely delivered groups were cheaper to deliver (average of £126.86) compared to face-to-face groups (average of £140.47), reduced due to no costs being required for room hire or refreshments.

The key cost drivers for delivering face-to-face Group Triple P differed between sites and this was primarily a consequence of the site’s funding model including their budget allocation to staffing, operational and overheads costs. The four sites chosen for this feasibility study differed in their scope and scale. Staff employed by the different sites broadly included social workers (of varying grades), intervention facilitators, primary school teachers and parenting practitioners. Individual site costs for delivery of Group Triple P depended on staff allocation to complete the various tasks required for implementation, particularly in terms of the level of staff seniority and the frequency and duration of time allocated to different activities.

#### An assessment of the broader resource use and health-related quality outcomes associated with delivering Group Triple P remotely or face-to-face

Broader health and social care resource use was collected from parents, using an adapted Client Service Receipt Inventory (CSRI) [31] at baseline and at 16-week follow-up timepoints. Resource use items were summarised by study arm using a complete case approach, where data was completed by families for costs and outcomes at baseline and follow-up. Group means and standard errors for resource use values by resource category were estimated. Supplementary material 1 presents the resource use data for parents and their children for the previous 16-week period, by trial arm and reported at baseline and follow-up. In the time period between baseline and follow-up, participants described the following child health and social care resource use: GP surgery contacts (0.36 contacts in the remote delivery arm and 0.20 in the face-to-face arm), social workers (0.14 contacts in the remote arm compared with 0.40 in the face-to-face arm), speech and language professionals (0.29 in the remote arm compared with 0.10 in the face-to-face arm), childcare (7.79 uses per child in the remote arm and 15.6 times in the face-to-face arm). Use of hospital services, such as inpatient, day case and outpatient services, was ‘low to none’ in both arms and low levels of medication use (no children in the remote arm used medication compared with 1 child in the face-to-face arm).

#### Identification of appropriate sources of unit costs for potential resource consequences

Unit costs were obtained from a variety of sources, to derive the most accurate estimates of cost data. These are shown in the supplementary material 2 and included costs extracted directly from published reports and inflated to current prices, and unit costs for community-based health and social services derived from routine sources such as the Compendium of the Unit Costs of Health and Social Care 2023, from the Personal Social Services Resource Unit (PSSRU) [40, 41]. Sources of unit costs for hospital admissions over the trial time horizon, inpatient care, outpatient care and accident and emergency visits, were obtained from national tariffs (NHS reference costs trusts schedules). The costs of home visits, calls to NHS direct, Child and Adolescent Mental Health Service (CAMHS) contacts and home care were derived from other sources including reports from The Kings Fund [42] and published reports for the NIHR Health Services and Delivery Research programme as referenced in the table below. Most of the unit costs shown in the table above were easily accessible from routine sources or published reports, and they are consistent across time when accounting for inflation. Unit costs that require primary micro-costing or updating from isolated reports include the costs of calls to NHS Direct, private nursery and childcare days and specialist support at school. For parents these also included other private care services.

#### Analysis of health-related quality-of-life outcomes for parents

For parents, health-related quality of life was assessed using the EQ-5D-5L data instrument where completions were obtained at baseline and 16 weeks follow-up [30]. Outcomes are presented as utility scores. The utility scores are indexed at 0–1. The utility measure summarises both the positive and negative effects of an intervention into values indexed at 0 and 1 (where 0 represents death, 1 represents full health and values below 0 indicate health states that are worse than death). The utility scores can be converted into quality-adjusted life-years (QALYs) using an area under the baseline-adjusted utility curve and can be calculated using linear interpolation between utility scores at baseline and 16 weeks. The EQ-5D-5L has two measurement components. The first component categorises health-related quality of life into the following five dimensions: (1) ‘mobility’, (2) ‘self-care’, (3) ‘usual activities’, (4) ‘pain/discomfort’ and (5) ‘anxiety/depression’. Participants report their level of function for each dimension within one of following five ordinal levels: (1) no problems, (2) slight problems, (3) moderate problems, (4) severe problems and (5) extreme problems/unable to perform. Parent responses were used to construct an EQ-5D-5L utility score for each respondent.

The second part of the EuroQol-5 Dimensions (EQ-5D) is a visual analogue scale (VAS), which ranges from 100 (best health condition) to 0 (worst health condition) and describes the participant’s own assessment of their health status on the day of completion. Health-related quality-of-life outcomes for parents were generally rated highly (i.e. higher than 50% or 70%) across all domains. They were marginally better for both groups at 16 weeks follow-up when compared to baseline. However, they were also marginally better for the remote group than for the face-to-face group, reflecting a greater improvement in the intervention group over time.

#### Analysis of health-related quality-of-life outcomes for children

The health-related quality of life of the children was assessed using a range of age-appropriate measures, where completions were obtained for both baseline and 16 weeks follow-up resulting in very small numbers. The CHU9D instrument is patient-reported outcome, a generic preference-based measure of health-related quality of life, a measure to generate Quality-Adjusted Life Years (QALYs) developed with children and originally validated for a target age of 7–17 years. It consists of nine dimensions: (1) ‘worried’, (2) ‘sad’, (3) ‘pain’, (4) ‘tired’, (5) ‘annoyed’, (6) ‘schoolwork/homework’, (7) ‘sleep’, (8) ‘daily routine’ and (9) ‘usual activities’, and each dimension is represented by a single question with five response options. The recall period is today/last night. The 9-item instrument can generate almost 2,000,000 health states (9 items, 5 levels each). The questions in the CHU9D have been adapted for younger children, and with parent-proxy report, though the dimensions and levels remain the same. It was found to be valid and reliable to measure health-related quality-of-life in children aged 2–4 and 5–6 years, although with relatively low test–retest reliability in some dimensions [32]. Two self-reported instruments were completed by children aged 8 and older. The first is the EQ-5D-Y-3L. The second self-reported instrument capturing outcomes and completed by children of years eight and older was the CHU9D. Quality-adjusted life years (QALYs) are able to be directly obtained from this instrument and age group for use in cost-utility analysis. Health-related quality-of-life outcomes for children of 3–5 years of age across all domains demonstrated that there were greater numbers of ‘less-then-optimal’ scores for ‘tiredness’, ‘ability to undertake usual routines’, and ‘sleep’. No children were reported to experience ‘pain’. There were no observable reductions in ‘worry’ or ‘schoolwork/homework’. Marginal improvements could be identified for both groups at 16 weeks follow-up when compared to baseline for ‘sadness’, ‘annoyed’, ‘sleep’, ‘daily routines’ and ‘health today’. Overall, reported ‘health today’ was marginally better for the remote group than for the face-to-face group.

#### An assessment of the best possible way of expressing cost-effectiveness

The primary research objective for an embedded economic evaluation within a future definitive trial would be to estimate the incremental cost-utility of delivering remote Group Triple P to parents at baseline and 16 weeks follow-up. Secondary outcomes include an estimation of parent health-related quality of life outcomes at baseline and 16 weeks follow-up. In the first instance, parent and child costs and outcomes would be reported separately, though methodological and modelling related approaches that combine the presentation of cost-effectiveness and/or cost-utility for a parent–child dyad should be explored. It is anticipated that QALY-based approaches are not yet sufficiently developed to capture all the disparate effects of Group Triple P for both parents and children, due to methodological challenges surrounding aggregation of disparate benefits for parents and children in a single metric. To combine disparate outcomes for parents and children in a single preference-based outcome measure will be a challenge for a future economic evaluation; however, a future trial would provide an excellent vehicle for research to collect and explore modelling approaches to examine this in terms of the parameter estimates required and the design of a model structure.

#### Available routine health and social data sources that could be used to complement and validate self-reported resource utilisation data in a future data linkage study

An economic evaluation embedded into a future trial should aim to estimate the broader resource use of delivering the intervention and should include of all of the ‘downstream’ resource use and costs generated across the trial’s lifespan, including primary, secondary and community care, and the costs of medications. Routine data can be used to validate and complement resource use data from trial data collection instruments. This enables researchers to collect a broader set of retrospective or prospective data items than would be identified in the health economic questionnaires completed by trial participants, especially where supplementing incomplete or missing resource use information. Resource use and clinical outcomes data can be extracted from routine data sources as part of clinical trials, providing the trial with the advantage of accessing relevant data without having to request it from patients or study participants. Linkage with routine data can provide information about the medical history of patients, when self-reported questionnaires provide insufficient information. Routine linked data can additionally be used for modelling parameters of costs when an economic analysis starts after patient or participant follow-up is completed. Thus, routine data can be used to inform long-term epidemiological models of disease or health status progression. Based on the advantages of using routine linked data and in view of the data that might be required to inform the incremental cost-effectiveness for a unit of change in health-related quality of life (and additionally changes in education and social care outcomes) for parents and children, a future trial-based economic evaluation could consider extracting key resource use items from a variety of datasets.

### The acceptability of collecting and analysing routine data within a definitive trial

The acceptability of collecting and analysing routinely collected hospital, school and social care data within a future trial from the perspective of parents/caregivers was determined. Firstly, participants were asked whether they were aware that researchers could request access to routine data. The level of awareness varied for each data type; 43.8%, 31.3% and 28.0% of participants reported that they were aware that researchers could request access to routinely collected hospital, school and child social care data respectively. Secondly, participants were asked how likely they would take part in a future trial whereby routine data was collected. Similarly, the levels of participants that reported that they would be unlikely to take part in a future trial requesting access to routine data varied as per data type; 25% of participants stated that they would be less likely to take part in a trial if access to parent or child routinely collected hospital data was requested, 12.5% of participants reported that they were less likely to take part in a trial if access to routinely collected school data was requested and 34.4% stated that they would be less likely to take part in a trial if access to routinely collected child social care data was requested (see Table 14).

**Table 14:** Participants’ awareness and views surrounding incorporation of routine data collection in a future definitive trial

	Parent hospital data	Child hospital data	Child school data	Child social care data
	(***n*** = 32)	(***n*** = 32)	(***n*** = 32)	(***n*** = 32)
	***n*** (%)	***n*** (%)	***n*** (%)	***n*** (%)
**Awareness that researchers can request access to this routine data?**				
No	17 (53.1)	N/A	22 (68.8)	23 (71.9)
Yes	14 (43.8)	N/A	10 (31.3)	7 (28.1)
Missing	1 (3.1)	N/A	0 (0)	0 (0)
**Views surrounding researchers requesting access to this data in a future definitive trial?**				
Not at all comfortable	4 (12.5)	3 (9.4)	1 (3.1)	2 (6.3)
Not very comfortable	3 (9.4)	5 (15.6)	5 (15.6)	1 (3.1)
No preference	12 (37.5)	9 (28.1)	12 (37.5)	18 (56.3)
Quite comfortable	8 (25.0)	9 (28.1)	6 (18.8)	5 (15.6)
Very comfortable	5 (15.6)	6 (18.8)	8 (25.0)	6 (18.8)
Missing	0 (0)	0 (0)	0 (0)	0 (0)
**The impact of requesting consent to collect routine data in a future definitive trial**				
Definitely less likely to take part	4 (12.5)	4 (12.5)	1 (3.1)	4 (12.5)
Slightly less likely to take part	4 (12.5)	4 (12.5)	3 (9.4)	7 (21.9)
No difference	22 (68.8)	22 (68.8)	26 (81.3)	13 (40.6)
Slightly more likely to take part	1 (3.1)	1 (3.1)	1 (3.1)	3 (9.4)
Definitely more likely to take part	1 (3.1)	1 (3.1)	1 (3.1)	5 (15.6)
Missing	0 (0)	0 (0)	0 (0)	0 (0)

### Local Authority and independent service provider willingness to participate in a definitive trial

In order to establish service provider willingness to participate in a potential definitive trial in the future, Local Authorities and providers of Triple P were contacted by email to complete a questionnaire. The questionnaire was completed by 14 sites delivering parenting programmes. Ten sites (71.4%) were experienced and regularly delivered Group Triple P Level 4 and 11 sites (79%) reported that they deliver other group-based parenting programmes other than Group Triple P. Of the sites delivering Group Triple P Level 4, 50% delivered only the face-to-face programme, 10% only delivered the programme remotely and 40% offered both delivery methods.

Sites were asked to report how likely they would agree to different aspects of a potential definitive trial. Most sites (60%) reported that they would likely offer a face-to-face and remote programme in each school term over the course of a year, but 20% reported that this was very unlikely, and 20% did not respond. Less than half of sites (40%) reported that they would be willing to randomise parents/caregivers to whether they received face-to-face or the remotely delivered programme, whilst 30% reported that they were very unlikely to participate in a trial involving randomisation (30% did not respond). Forty percent of sites reported that they would likely be willing to restrict access to Group Triple P to only parents/caregivers who were trial participants, 40% reported that they would be unlikely to agree to this and 20% of sites did not respond. Fifty percent of sites reported that they would likely be willing to allow a researcher to observe sessions, 30% reported that they would be unlikely to agree to this and 20% did not respond. However, fewer sites would likely be willing to record the sessions and provide access to the trial team (40%). Finally, 40% of sites reported that they would likely be willing to restrict access to Group Triple for parents potentially randomised to a potential control arm in a future definitive trial, whilst 40% reported that they would be unlikely to agree with this and 20% did not respond.

### Assessment of fulfilment of progression criteria to effectiveness RCT

Traffic-light assessment against pre-defined progression criteria to a future definitive trial is described in Table 15. Due to amber allocations, modifications to participant recruitment and data collection processes would be essential for a future successful trial. Whilst many trial design features were feasible, inclusion of individual randomisation in a future trial design would not be acceptable to participants and intervention delivery site staff.

**Table 15:** Assessment of fulfilment of progression criteria

	Pre-defined progression criteria			Trial team Assessment
	Red *(Do not proceed to full trial)*	Amber *(Proceed to full trial with modifications)*	Green *(Proceed to full trial)*	
**Study recruitment**				
Number of approached parents/caregivers participating	< 40%	40–49%	> 50%	**Amber**
Recruitment target met	< 60%)	60–79%	> 80%	**Amber**
Consideration of the inclusion of underserved groups within the sample (e.g. fathers, parents/caregivers from ethnic minorities and differential drop-out of fathers in the remote delivery arm compared to the face-to-face arm				**Amber**
**Data collection**				
Collection of follow-up data from participants across all data collection methods, including remote methods	< 60%)	60–79%	> 80%	**Amber**
Assessment of the feasibility of collecting outcome data required for a future economic evaluation				**Green**
**Trial processes**				
Assessment of the acceptability of trial processes to participants and site staff				**Red/Amber**

## Discussion

There is growing interest in remote delivery of group-based parenting interventions, including its potential to address recruitment challenges and reduce barriers to attendance among underserved populations. However, research on feasibility, acceptability to practitioners and parents and effectiveness is limited [15–17]. Our study aimed to contribute to the emerging evidence in this area by evaluating the feasibility of conducting a randomised controlled trial comparing face-to-face and remote delivery of the Group Triple P parenting intervention.

The principal finding of this study was that a randomised controlled trial, in its current design, comparing face-to-face and remote delivery of the intervention would not be feasible. Recruitment of sites was hindered by the practical challenges of running face-to-face and remote groups simultaneously, and because randomisation undermined practitioners’ emphasis on preserving parental choice of intervention and delivery format. Despite being initially included in the study design, randomisation was not feasible in any of the sites, since it threatened to undermine the ability of practitioners to recruit sufficient numbers of participants to form two groups. Although data collection procedures, including the administration of questionnaires to children, were acceptable to participants, a substantial proportion of parents who consented to the study did not subsequently complete baseline measures. It is unclear why parents who consented to take part in the study did not go on to complete the baseline measures; however, more in-depth site training for staff recruiting participants to the study may have improved baseline data completion rates. Low data completion rates may also have been a consequence of remote data completion and a lack of relationship with or accountability to the researchers. Participants described several barriers to completing the trial data including the time taken to complete the questionnaires, technical difficulties being able to save progress on online surveys, and instructions. All of these challenges are surmountable for a future trial. Furthermore, the introduction of participant-selection of allocation arm, rather than by the trial team meant that sites were able to continue with arrangements for intervention attendance without prior completion of consent and baseline data collection, ultimately resulting in a mixed group of participants and non-participants attending the intervention group sessions. An adapted recruitment and allocation process, involving members of the research team responsible for recruitment, baseline data collection and ‘approval’ of allocation may prevent this in a future trial.

Remote delivery of Group Triple P was introduced in 2020 as an emergency response to COVID-19 restrictions and was initially considered an experimental approach. By the time the study commenced, however, many sites—including those participating—had integrated remote delivery as a permanent component of routine practice, which substantially reduced the feasibility and acceptability of randomising participants to different delivery formats. Remote delivery was often used to increase flexibility and parental choice, for example by offering face-to-face sessions during the day and remote sessions in the evening. Process evaluation findings suggested that, whilst many parents preferred face-to-face delivery, practical considerations such as timing and availability might lead them to opt for the remote format. This was reflected in the fact that our adoption of a non-randomised allocation process, whereby practitioners and parents jointly determined the delivery mode the latter received, produced a fairly even distribution between the two formats (23 remote, 19 face-to-face).

The demographic characteristics of participating families and children’s SDQ scores were comparable to those reported in other studies evaluating Group Triple P [43–45]. Overall, we found few differences in the profile of participants between the two study arms. Remote delivery did not appear to achieve greater involvement of underserved groups. Rates of participation by fathers and ethnic minority groups were low in both cases. The majority of participants were of white ethnic groups and 7.1% of the participants of non-white ethnic groups (Asian or Asian British—Indian or Pakistani, Black or Black British—Caribbean). This is lower than seen in the general population, suggesting that more work is required to ensure representative inclusion in future research. However, whilst rates of educational status were broadly comparable with the general population, rates of employed or self-employed participants observed (59.5%) were lower than that seen in the general population (75.0%). Levels of education were higher in the remote delivery arm, but it was not clear why this was the case. However, the low numbers of participants mean that caution must be taken when interpreting the baseline demographic results. There was, however, variation in recruitment rates and the recruitment strategies used across sites, which may have affected the reach of the intervention across sites. Furthermore, sites used established recruitment strategies; efforts to increase reach of parenting interventions among underserved groups and fathers may need to be linked to a broader set of recruitment strategies.

Implementation of the intervention using remote delivery was found to be feasible. Attendance was good across both arms, with a slightly higher proportion in the remote arm attending at least six of the eight sessions. Fidelity of remote delivery was high overall, with the structure and content being implemented consistently across sites. However, practitioners did make some adaptations in the delivery of activities for remote delivery, which could be incorporated into intervention delivery guidance These included extending the length of some sessions to ensure all content was covered, modifying role playing activities, or altering the final group session (week 8) to be a one-to-one telephone call if attendance had been very low. Careful consideration of implementation adaptations for remote delivery and optimisation of the intervention logic model would be required for any future research; these adaptations would need to be considered for intervention implementation, including the delivery manual, in a future trial.

Whilst practitioners valued the remote delivery format, they described several challenges, particularly in relation to participant engagement. These findings echo those of previous studies which have indicated that the transfer of group-based interventions to remote delivery creates challenges around how participants engage with the material presented and with other members of their group [15, 19, 20]. We found that whilst practitioners were able to deliver key content and interact with participants, it was often challenging to promote group interaction and learning (which have been identified as important intervention mechanisms). However, practitioners developed strategies to overcome such challenges (and to promote group interaction) such as use of breakout rooms. When interventions are designed with causal mechanisms that are expected to operate through group interaction, such strategies may be particularly important to ensuring the interventions work as intended. An important consideration in remote delivery would also be to provide remote safeguarding training to practitioners to help them navigate issues that are specific to remote delivery.

Our study adds to the limited research base on the feasibility and acceptability of remote delivery of group-based parenting interventions. Similar to previous work in this area, we found that intervention implementation was feasible [17]. We addressed the limitations of earlier work (noted in Fang et al.’s [17] systematic review) by collecting detailed data on fidelity, which indicated that the programme had been delivered as intended. We also found that remote delivery was broadly acceptable to practitioners and parents, though it did not appear to systematically increase reach among underserved groups (again, mirroring the findings of previous studies [22, 23]. One of the key implications of our findings is the importance of understanding contextual factors in shaping parental preference for delivery method. Use of remote delivery by intervention providers as part of an integrated set of options to maximise parental choice and flexibility means that it will likely sit alongside face-to-face provision, rather than as a standalone initiative to achieve cost efficiencies or increase reach. This is an important direction for future research.

Routine data is a useful tool in research trials and the extraction of routinely collected data from various sources including the NHS, schools and social care, can offer research data without requiring data completion by participants. However, the collection of routinely collected data requires informed participant consent. Our findings suggest that between 25 and 34.4% of participants would not feel comfortable with researchers accessing these types of routine data. Although this apprehension may be overcome by educating participants regarding data security, this is a significant barrier to using routinely collected data in a definitive trial. Further qualitative work with parents should focus on understanding and overcoming parents’ specific concerns.

Our study has several strengths. Use of a mixed methods design, including process and economic evaluations, enabled us to identify key factors affecting feasibility of a future RCT and describe how they operated. Process evaluation data on recruitment, implementation fidelity and intervention adherence by participants enabled us to provide a detailed ‘real world’ account of the feasibility of remote delivery. A number of limitations should also be noted. The eventual use of a non-randomised design for participant recruitment meant that there were specific aspects of the feasibility of an RCT design that we could not assess (e.g. participant retention rates across randomised arms). Although there was variation in the characteristics of the four sites (e.g. rural/urban locations, delivery structures), there are limitations in the extent to which findings can be generalised to all settings across the UK or internationally. The small sample size achieved also places limitations on the extent to which variation between face-to-face and remote groups (e.g. demographic profiles) could be established.

## Conclusions

In this study, we found evidence to support the feasibility and acceptability of remote delivery of the Group Triple P parenting intervention. The study identified that parents’ preferences regarding delivery method were shaped by multiple influences, including the value that they placed on face-to-face interaction and the flexibility which remote delivery sometimes offered. Whilst implementation of remote delivery was feasible, practitioners reported that participants sometimes interacted primarily with the intervention material, with reduced engagement in group interaction.

It would not be feasible to proceed with a randomised controlled trial using the design adopted in this study to assess the effectiveness of remote delivery of Group Triple P. Factors limiting feasibility related mainly to the way in which remote delivery has moved relatively quickly from being an experimental approach to an integrated aspect of routine practice, and its use to maximise flexibility and parental choice on intervention receipt.

Our findings highlight the importance of ongoing research to assess the use of remotely delivered group-based parenting interventions. In particular more evidence is needed on their reach among underserved groups across different contexts and different intervention types. The design and implementation of group-based interventions which explicitly aim to promote group process as part of their hypothesised change mechanisms, also warrants further attention. Our feasibility study encountered a number of barriers to using an RCT design to evaluate a remotely delivered version of an existing intervention that was already well-established using face-to-face delivery. Evaluation of development and adaptation of interventions for remote delivery can build knowledge concerning how best to design them, and whether they are more appropriate in particular settings. It is important that we identify strategies to overcome the identified challenges or consider potential non-randomised definitive trial or cluster randomised-controlled trial designs, thus ensuring that important innovations in the delivery of parenting (and other public health) interventions are rigorously evaluated to assess their effectiveness.

## Supplementary Information

Supplementary Material 1.

Supplementary Material 2.

## Data Availability

The datasets used and/or analysed during the current study are available from the corresponding author on reasonable request.

## Abbreviations

ALPHA: Advice Leading to Public Health Advancement
APS: Arnold and O’Leary Parenting Scale
CAMHS: Child and Adolescent Mental Health Service
CHEERS: Consolidated Health Economic Evaluation Reporting Standards
CHU9D: Child Health Utility 9 Dimension Instrument
CONSORT: Consolidated Standards of Reporting Trials
CSRI: Client Service Receipt Inventory
DASS: Depression Anxiety Stress Scale
DECIPHer: Centre for Development, Evaluation, Complexity and Implementation in Public Health Improvement
EQ-5D: EuroQol-5 Dimensions
GP: General Practitioner
MRC: Medical Research Council
NHS: National Health Services
PAFAS: Parenting and Family Adjustment Scales
PAG: Parent’s Advisory Group
PROMS: Participant-reported outcome measures
QALYs: Quality-adjusted life-years
QAP: Qualitative analysis plan
RCT: Randomised controlled trial
SDQ: Strengths and Difficulties questionnaire
SHEAP: Statistical and health economics analysis plan
SPIRIT: Standard Protocol Items: Recommendations for Interventional Trials
SSC: Study Steering Committee
TPUK: Triple P UK
VAS: Visual analogue scale
