# Supplementary material for: Remote versus face-to-face delivery of the Group Triple P parenting programme: a feasibility non-randomised trial
Source: Pilot Feasibility Stud. 2026 Jun 24;12:100. doi: 10.1186/s40814-026-01861-3 (PMC13374339; doi:10.1186/s40814-026-01861-3)
Supplement: Supplementary file 1 — Supplementary Material 1. [file 40814_2026_1861_MOESM1_ESM.docx]

*Supplementary material 1. Resource use values for families with complete data by trial allocation and trial period at baseline and 16-weeks follow-up (£, 2024)*

|  | **Baseline** | | **Follow-up** | |
| --- | --- | --- | --- | --- |
|  | Remote | Face-to-face | Remote | Face-to-face |
| Resource use | n=14 | n=10 | n=14 | n=10 |
| **Child** |  |  |  |  |
| **Health care, social care, community or private service, mean (SE)** | | | | |
| GP surgery (contacts) | 0.64 (0.37) | 0.10 (0.10) | 0.36 (0.19) | 0.20 (0.13) |
| GP out of hours (contacts) | 0.07 (0.07) | 0.00 (0.00) | 0.00 (0.00) | 0.00 (0.00) |
| GP home visit (contacts) | 0.00 (0.00) | 0.00 (0.00) | 0.00 (0.00) | 0.00 (0.00) |
| General practice nurse (contacts) | 0.36 (0.36) | 0.10 (0.10) | 0.00 (0.00) | 0.00 (0.00) |
| Calls to NHS direct (contacts) | 0.28 (0.16) | 0.10 (0.10) | 0.00 (0.00) | 0.10 (0.10) |
| District nurse (contacts) | 0.00 (0.00) | 0.00 (0.00) | 0.00 (0.00) | 0.00 (0.00) |
| Health visitor (contacts) | 0.21 (0.15) | 0.00 (0.00) | 0.00 (0.00) | 0.00 (0.00) |
| Social worker (contacts) | 0.14 (0.14) | 0.30 (0.30) | 0.14 (0.14) | 0.40 (0.40) |
| Paediatrician (contacts) | 0.07 (0.07) | 0.00 (0.00) | 0.00 (0.00) | 0.00 (0.00) |
| Ophthalmologist (contacts) | 0.07 (0.07) | 0.10 (0.10) | 0.07 (0.07) | 0.10 (0.10) |
| Audiologist(contacts) | 0.00 (0.00) | 0.00 (0.00) | 0.00 (0.00) | 0.00 (0.00) |
| Speech and language (contacts) | 0.07 (0.07) | 0.00 (0.00) | 0.29 (0.29) | 0.10 (0.10) |
| Clinical Psychologist (contacts) | 0.00 (0.00) | 0.00 (0.00) | 0.00 (0.00) | 0.00 (0.00) |
| Educational Psychologist (contacts) | 0.00 (0.00) | 0.00 (0.00) | 0.00 (0.00) | 0.00 (0.00) |
| Psychiatrist (contacts) | 0.00 (0.00) | 0.00 (0.00) | 0.00 (0.00) | 0.00 (0.00) |
| CAHMS (contacts) | 0.00 (0.00) | 0.00 (0.00) | 0.00 (0.00) | 0.00 (0.00) |
| Dentist (contacts) | 0.21 (0.11) | 0.50 (0.31) | 0.29 (0.12) | 1.00 (0.36) |
| Dentist (telephone contacts) | 0.07 (0.07) | 0.40 (0.30) | 0.00 (0.00) | 0.80 (0.59) |
| Positive Behavioural Support team (contacts) | 0.00 (0.00) | 0.00 (0.00) | 0.00 (0.00) | 1.60 (1.60) |
| Occupational therapist (contacts) | 0.00 (0.00) | 0.00 (0.00) | 0.00 (0.00) | 0.00 (0.00) |
| Private nursery (days) | 0.57 (0.57) | 14.4 (7.71) | 7.79 (5.38) | 15.6 (8.52) |
| Additional support in school (contacts) | 0.14 (0.14) | 0.80 (0.80) | 0.00 (0.00) | 0.40 (0.40) |
| Other health services (contacts) | 0.07 (0.07) | 0.00 (0.00) | 0.00 (0.00) | 0.10 (0.10) |
| Other social care services (contacts) | 0.29 (0.29) | 0.40 (0.40) | 0.00 (0.00) | 0.00 (0.00) |
| **Hospital or day services, mean (SE)** |  |  |  |  |
| Hospital inpatient stay (days) | 0.00 (0.00) | 0.10 (0.10) | 0.00 (0.00) | 0.00 (0.00) |
| Hospital day centre (visits) | 0.00 (0.00) | 0.10 (0.10) | 0.00 (0.00) | 0.00 (0.00) |
| Accident and emergency (visits) | 0.00 (0.00) | 0.00 (0.00) | 0.00 (0.00) | 0.00 (0.00) |
| Outpatient clinic (visits) | 0.07 (0.07) | 0.10 (0.10) | 0.00 (0.00) | 0.00 (0.00) |
| Day development centre (visits) | 0.14 (0.14) | 0.00 (0.00) | 0.00 (0.00) | 0.00 (0.00) |
| Medication use, n (%) | 2 (14.3) | 1 (10) | 0 (0) | 1 (10) |
| **Parents** | | | | |
| **Health care, social care, community or private service, mean (SE)** | | | | |
| GP surgery (contacts) | 0.36 (0.22) | 0.70 (0.21) | 0.29 (0.22) | 0.90 (0.58) |
| GP surgery (telephone contacts) | 1.07 (0.65) | 0.90 (0.28) | 1.57 (1.42) | 0.00 (0.00) |
| GP out of hours (contacts) | 0.00 (0.00) | 0.00 (0.00) | 0.00 (0.00) | 0.00 (0.00) |
| GP home visit (contacts) | 0.00 (0.00) | 0.00 (0.00) | 0.00 (0.00) | 0.00 (0.00) |
| General practice nurse (contacts) | 0.07 (0.07) | 0.20 (0.13) | 0.07 (0.07) | 0.20 (0.13) |
| Calls to NHS direct (contacts) | 0.00 (0.00) | 0.00 (0.00) | 0.07 (0.07) | 0.00 (0.00) |
| District nurse (contacts) | 0.00 (0.00) | 0.00 (0.00) | 0.00 (0.00) | 0.00 (0.00) |
| Health visitor (contacts) | 0.00 (0.00) | 0.10 (0.10) | 0.00 (0.00) | 0.00 (0.00) |
| Social worker (contacts) | 0.14 (0.14) | 0.30 (0.30) | 0.14 (0.14) | 0.40 (0.40) |
| Physiotherapist (contacts) | 0.07 (0.07) | 0.00 (0.00) | 0.14 (0.09) | 0.00 (0.00) |
| Psychologist | 0.00 (0.00) | 0.00 (0.00) | 0.00 (0.00) | 0.00 (0.00) |
| Psychiatrist (contacts) | 0.00 (0.00) | 0.00 (0.00) | 0.00 (0.00) | 0.00 (0.00) |
| Dentist | 0.14 (0.09) | 0.40 (0.22) | 0.14 (0.14) | 0.50 (0.16) |
| Dentist (telephone contacts) | 0.14 (0.09) | 0.20 (0.13) | 0.00 (0.00) | 0.30 (0.15) |
| Counsellor (contacts) | 0.43 (0.43) | 0.00 (0.00) | 0.35 (0.35) | 0.00 (0.00) |
| Other social service support (contacts) | 0.14 (0.09) | 0.00 (0.00) | 0.00 (0.00) | 0.00 (0.00) |
| **Hospital or day services, mean (SE)** |  |  |  |  |
| Hospital inpatient stay (days) | 0.00 (0.00) | 0.00 (0.00) | 0.07 (0.07) | 0.00 (0.00) |
| Hospital day centre (visits) | 0.00 (0.00) | 0.00 (0.00) | 0.07 (0.07) | 0.00 (0.00) |
| Accident and emergency (visits) | 0.00 (0.00) | 0.10 (0.10) | 0.07 (0.07) | 0.00 (0.00) |
| Outpatient clinic (visits) | 0.00 (0.00) | 0.20 (0.20) | 0.00 (0.00) | 0.00 (0.00) |
| Other hospital service (visit) | 0.07 (0.07) | 0.00 (0.00) | 0.00 (0.00) | 0.00 (0.00) |
| Medication use, n (%) | 3 (21.4) | 5 (50) | 2 (14.3) | 3 (30) |
