# Supplementary material for: Remote versus face-to-face delivery of the Group Triple P parenting programme: a feasibility non-randomised trial
Source: Pilot Feasibility Stud. 2026 Jun 24;12:100. doi: 10.1186/s40814-026-01861-3 (PMC13374339; doi:10.1186/s40814-026-01861-3)
Supplement: Supplementary file 2 — Supplementary Material 2. [file 40814_2026_1861_MOESM2_ESM.docx]

Supplementary material 2: Unit costs associated with Resource Use (data available upon request)

|  | **Unit cost or range (£)** | **Source of unit cost** | **Source of unit cost, additional details** |
| --- | --- | --- | --- |
| **Health care, social care voluntary or private services** | | | |
| GP surgery (contact face-to-face) | 49 | PSSRU 2022/23^64^ | Table 9.4.2 |
| GP Surgery (e-consultation) | 43.92 | PSSRU 2022/23^66^ | Table 9.5.1 |
| GP Surgery (telephone triage) | 20.27 | PSSRU 2022/23^66^ | Table 9.6.1 |
| GP home per visit (visit) | 56 -200 | The Kings Fund 22/23 | Key facts and figures about the NHS. https://www.kingsfund.org.uk/insight-and-analysis/data-and-charts/key-facts-figures-nhs |
| General practice nurse (hour) | 53 | PSSRU 2022/23^62^ | Table 9.3.1 |
| GP Practice Nurse (contact telephone triage) | 9.04 | PSSRU 2022/23^67^ | Table 9.6.1 |
| Calls to NHS direct (contact) | 11.40 | Impact of NHS 111 Online on the NHS 111 telephone service and urgent care system: a mixed-methods study. | Health Services and Delivery Research, No. 9.21.  Turner J, Knowles E, Simpson R, et al.  Southampton (UK): [NIHR Journals Library](http://www.journalslibrary.nihr.ac.uk/hsdr);  https://www.ncbi.nlm.nih.gov/books/NBK575169/ |
| District nurse band 7 (hour) | 74 | PSSRU 2022/23^61^ | Table 9.2.1 |
| Health visitor - nurse band 4 (hour) | 44 | PSSRU 2022/23^61^ | Table 9.2.1 |
| Paediatrician (contact face-to-face) | 143 | PSSRU 2022/23^95^ | Table 11.3.2 |
| Ophthalmology (contact) | 143 | PSSRU 2022/23^95^ | Table 11.3.2 |
| Audiology Band 8c (contact) | 96 | PSSRU 2022/23^88^ | Table 11.1.2 |
| Speech and language Band 6 (contact) | 50 | PSSRU 2022/23^88^ | Table 11.1.2 |
| Clinical Psychologist Band 8D (hour) | 115 | PSSRU 2022/23^88^ | Table 11.1.2 |
| Educational Psychologist (hour) | 96 | PSSRU 2022/23^88^ | Table 11.1.2 |
| Psychiatrist (hour) | 143 | PSSRU 2022/23^95^ | Table 11.3.2 |
| Dentist (hour)^a^ | 222 | PSSRU 2022/23^69^ | Table 9.8.1 |
| Child and adolescent mental health service provider (Contact) | 34 to 141 | Mental health 360: funding and costs  21 February 2024 | https://www.kingsfund.org.uk/insight-and-analysis/long-reads/mental-health-360-funding-costs |
| Physiotherapist band 7 (hour) | 63 | PSSRU 2022/23^56^ | Table 8.2.1 |
| Positive behaviour support therapist (hour) | 21 | PSSRU 2022/23^69^ | Table 2.5.1 |
| Occupational therapist Band 6 (hour) | 53 | PSSRU 2022/23^56^ | Table 8.2.1 |
| Private nursery (week) | 236.36 | Childcare Costs Survey 2024 | <https://www.daynurseries.co.uk/advice/childcare-costs-how-much-do-you-pay-in-the-uk> (Berg, 2024a) <https://www.daynurseries.co.uk/advice/childcare-costs-how-much-do-you-pay-in-the-uk> (Berg, 2024) |
| After school care (week) | 69.14 | Childcare Costs Survey 2024 | <https://www.daynurseries.co.uk/advice/childcare-costs-how-much-do-you-pay-in-the-uk> (Ber, 2024b) |
| 1:1 care (week) | 400 - 640 | Childcare Costs Survey 2024 | <https://www.daynurseries.co.uk/advice/childcare-costs-how-much-do-you-pay-in-the-uk> |
| Other health or community care support (contacts) | 31 | PSSRU 2022/23^80^ | Table 10.6.1 |
| Social care services (social worker) (hour)^b^ | 53 | PSSRU 2022/23^75^ | Table 10.1.1 |
| Voluntary care services (contact) | 27 | PSSRU 2022/23^78^ | Table 10.4.1 |
| Private care services (contact) | 24 - 50 | Paying for care at home survey - 2024 | <https://www.homecare.co.uk/advice/paying-for-care-at-home> |
| Home help (hour) | 27 | PSSRU 2022/23^75^ | Table 10.4.1 |
| Counsellor Band 6 (hour) | 53 | PSSRU 2022/23^80^ | Table 8.2.1 |
| **Hospital services** | | | |
| Hospital inpatient stay (day) | 413.72 | NHS reference costs 2022/23 | APC - Regular Day or Night Admissions  Admission without Interventions, with CC Score 1+ |
| Hospital day centre (visit) | 651.77 | NHS reference costs 2022/23 | APC - Regular Day Admissions  Admission without Interventions |
| Hospital accident and emergency (visit) | 164.69 | NHS reference costs 2022/23 | EC: minor injury activity type in A&E/with reception of accident and emergency patients. VB01Z 03 |
| Hospital outpatient’s clinic (visit) | 186.8 | NHS reference costs 2022/23 | Community Service AA32Z 290 |
